# A Split-Mouth Comparison Between Platelet-Rich Plasma and Lymphomyosot for Management of the Postoperative Sequelae After the Extraction of Impacted Third Molars: A Case Report

**DOI:** 10.7759/cureus.74646

**Published:** 2024-11-28

**Authors:** Ralitsa V Yotsova

**Affiliations:** 1 Department of Oral Surgery, Medical University of Varna, Varna, BGR

**Keywords:** facial edema, lymphomyosot, pain, platelet-rich plasma, post-extraction sequelae, third molar extraction, trismus

## Abstract

Third molar extraction is one of the most common interventions in oral surgery. It is usually associated with postoperative pain, edema, and trismus. The severity of these sequelae can be related to the amount of surgical trauma and the duration of the extraction. Prevention strategies, including different local and systemic medications, autologous platelet concentrates, and physical therapy, can be beneficial for reducing postoperative discomfort and risk of complications.

This case report compares the local application of platelet-rich plasma (PRP) and the systemic use of the homeopathic combination medication Lymphomyosot to reduce the postoperative sequelae after third molar extraction. The study results revealed the superior qualities of PRP in reducing postoperative edema and trismus, while both methods gave similar results regarding pain control.

## Introduction

The extraction of impacted third molars involves full-thickness flap reflection and alveolar bone fenestration, which can lead to some postoperative sequelae, such as pain, edema, and trismus [1]. The severity of these symptoms depends on the amount of surgical trauma and the duration of the procedure [2]. In addition, some individual characteristics, such as gender, weight, and body mass index, have also been reported to influence the healing process and postoperative edema [3,4].

Many patients report functional disorders and deterioration in the quality of life in the immediate post-surgical period. Therefore, prevention strategies are necessary to reduce the risk of complications, postoperative discomfort, and the number of follow-up visits. These strategies include, but are not limited to careful planning; an exact surgical technique; medication with antibacterial drugs, corticosteroids, non-steroidal anti-inflammatory drugs, and local antiseptics; cryotherapy; laser therapy; and so on [5,6].

This case report compares the local application of platelet-rich plasma (PRP) and the systemic use of the homeopathic combination medication Lymphomyosot as methods aimed at reducing postoperative pain, edema, and trismus.

## Case presentation

**Figure 1:**
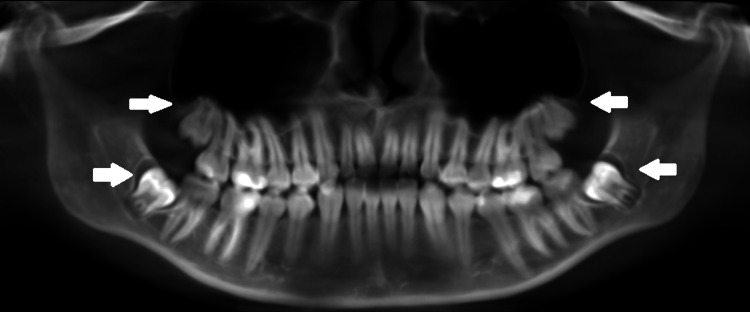
Orthopantomogram generated from the CBCT. White arrows indicating wisdom teeth impaction. CBCT: cone-beam computed tomography

A 17-year-old female patient was referred to the University Medical and Dental Centre at the Medical University of Varna, Bulgaria, at the end of August 2024 for extraction of her wisdom teeth before orthodontic therapy. The patient had no comorbidities, did not take systemic medication, and had no diagnosed allergies. The physical and radiological examination revealed fully impacted third molars without any signs of inflammation and/or infection (Figure 1). The patient and her mother insisted that the procedure was done under sedation due to the extreme fear of the patient and unwillingness to have her teeth extracted under local anesthesia. The patient was consulted by an anesthesiologist who referred her for standard blood and allergological testing, and the first surgical intervention for extraction of FDI teeth #18 and 48 was scheduled. A written informed consent was obtained from the legally authorized representative (patient’s mother) in accordance with the Declaration of Helsinki.

First surgical intervention

**Figure 2:**
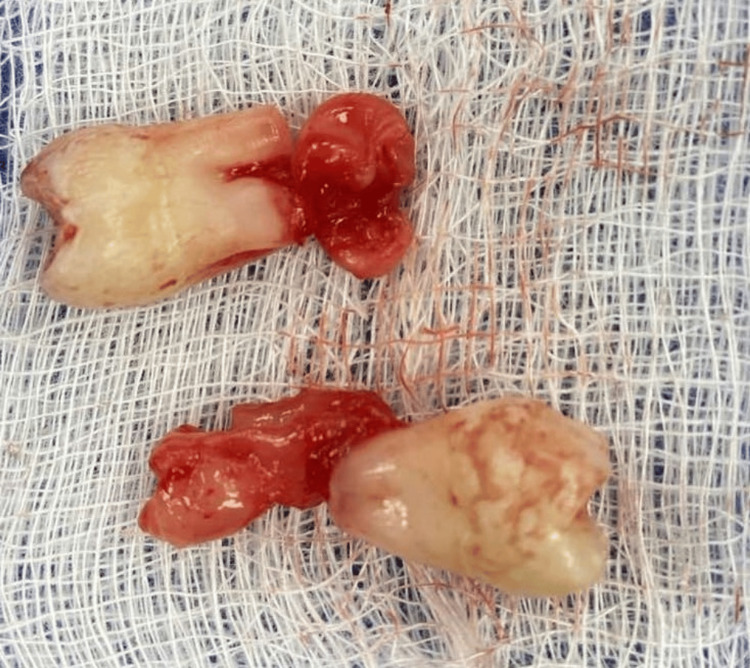
Extracted teeth #18 and #48.

**Figure 3:**
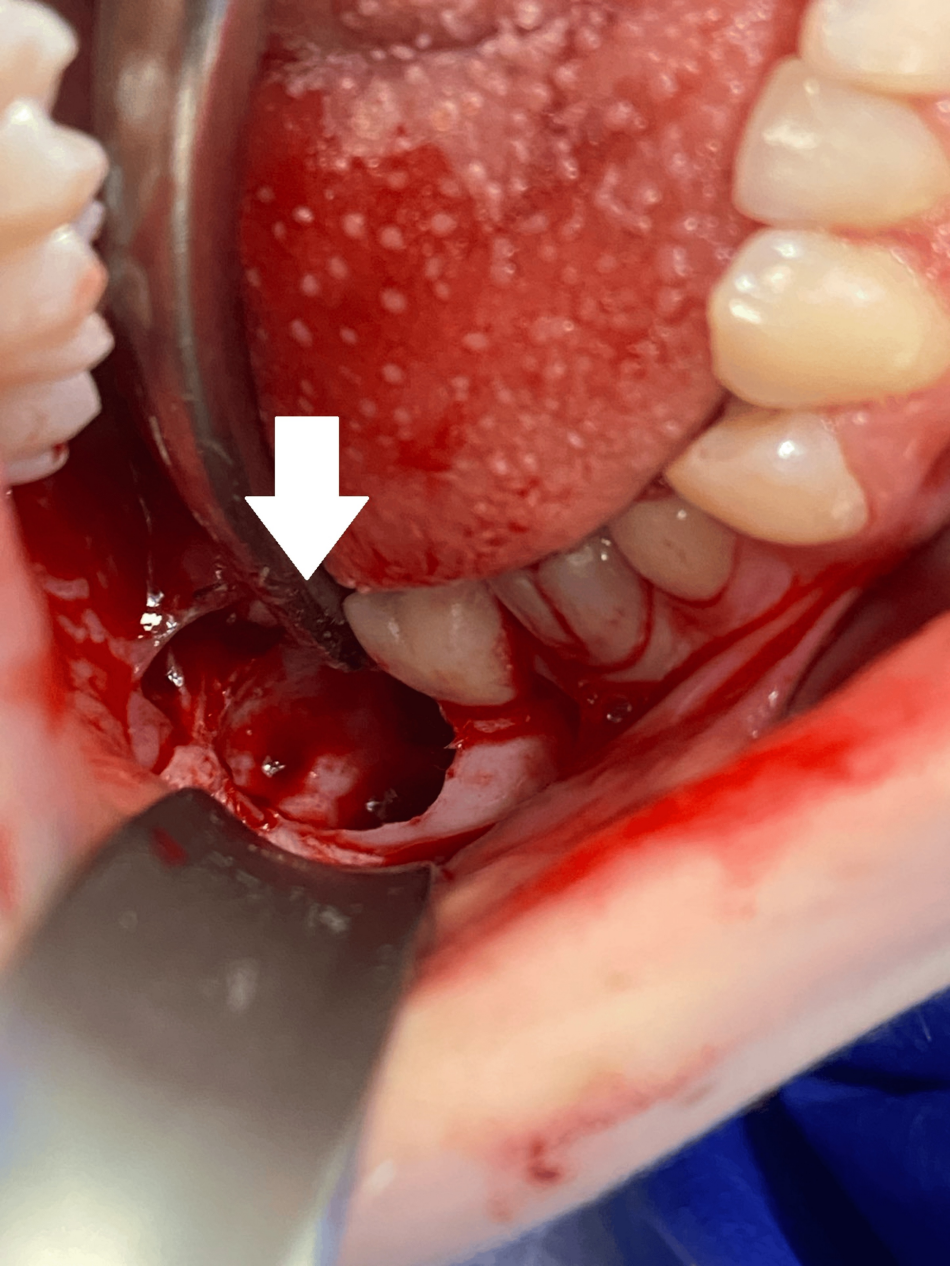
Post-extraction socket after the removal of tooth #48.

**Figure 4:**
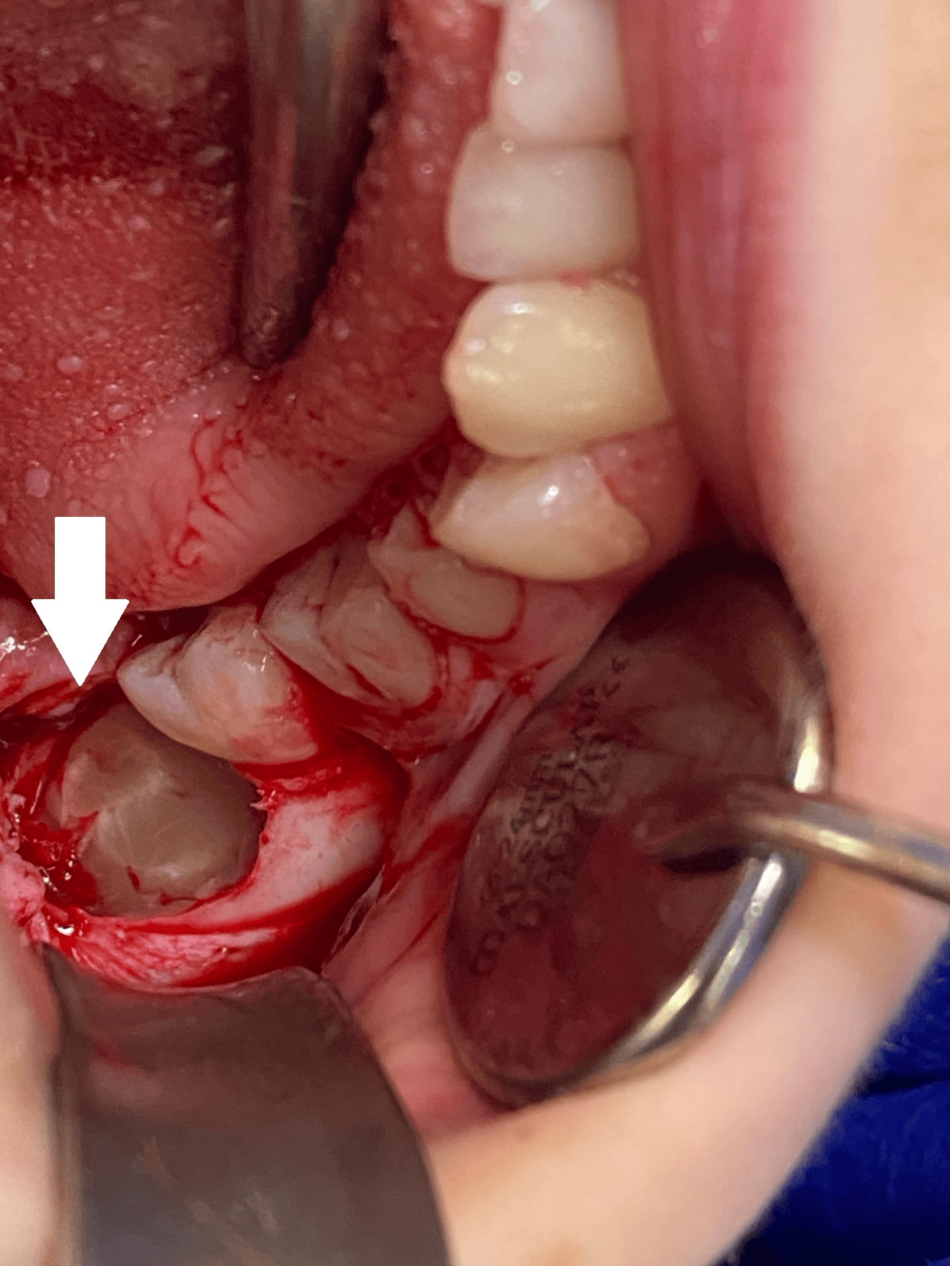
Post-extraction socket of tooth #48, filled with PRP. PRP: platelet-rich plasma

**Figure 5:**
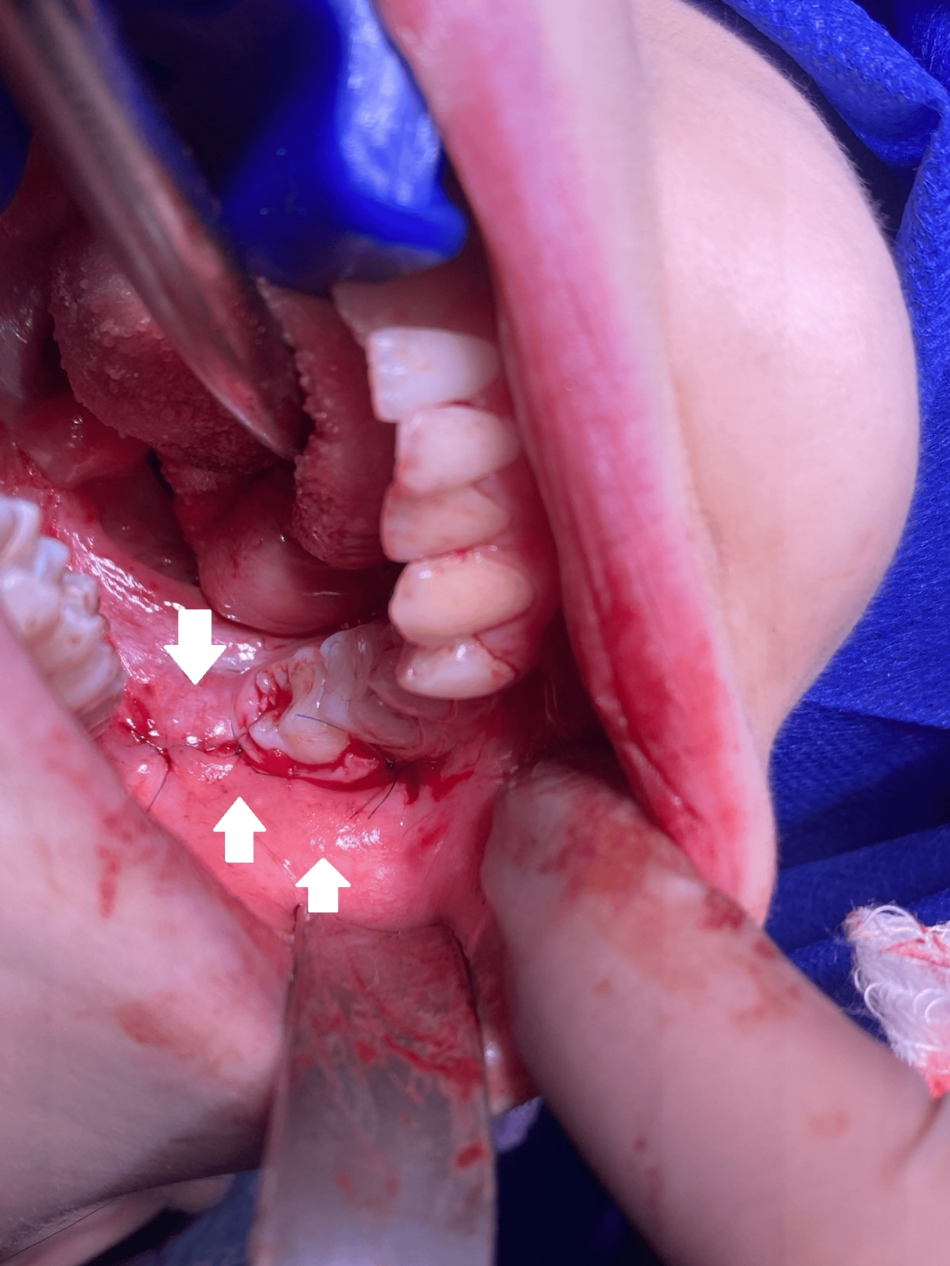
Repositioned and sutured flap over the socket of tooth #48.

**Figure 6:**
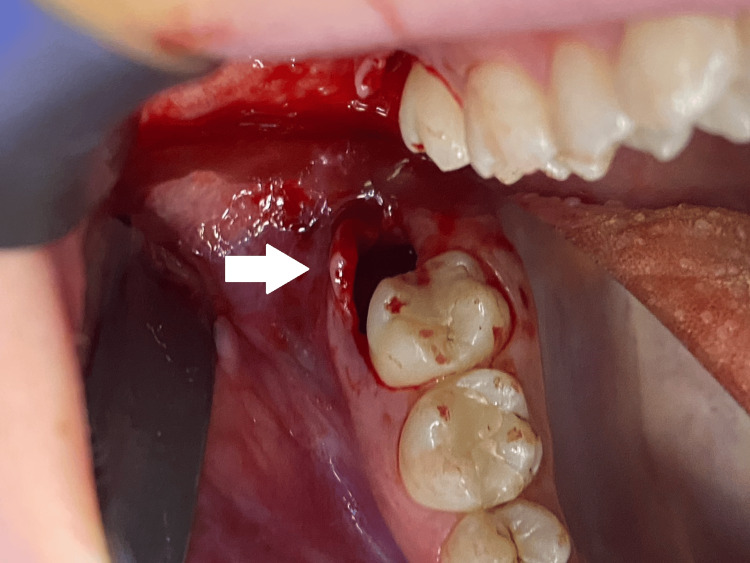
Post-extraction socket after the removal of tooth #18.

**Figure 7:**
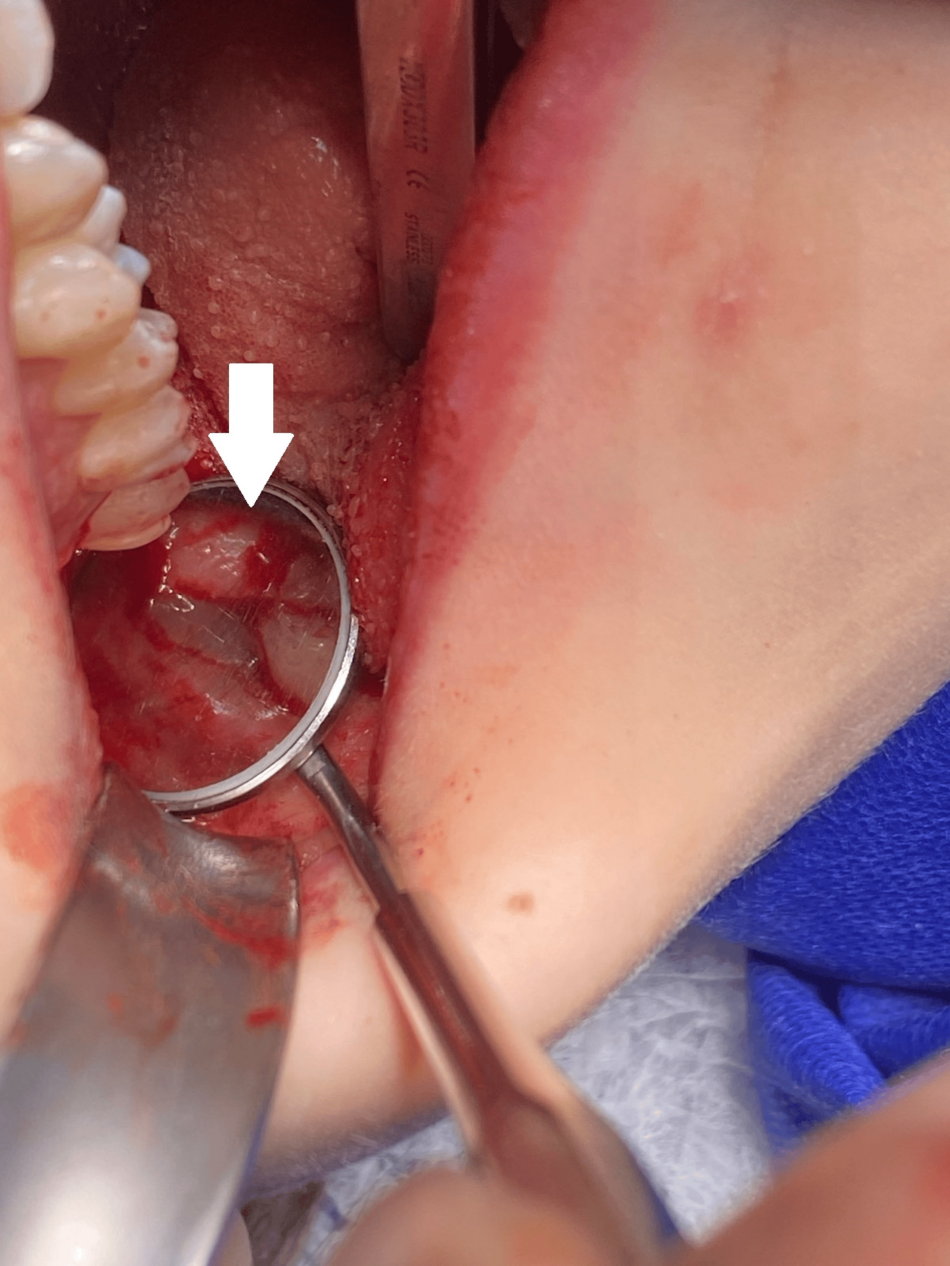
Post-extraction socket of tooth #18, filled with PRP. PRP: platelet-rich plasma

**Figure 8:**
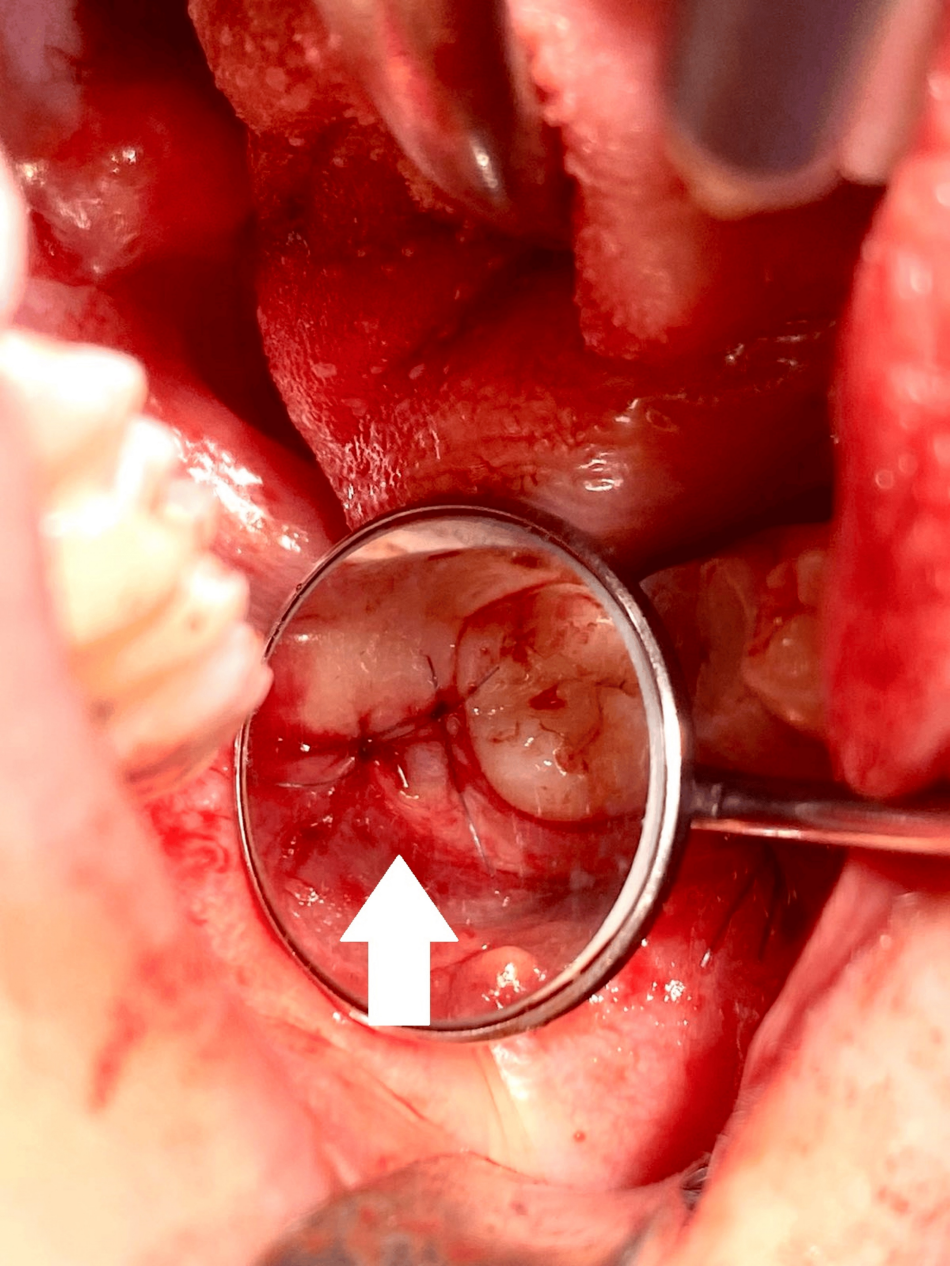
Repositioned and sutured flap over the socket of tooth #18.

At the beginning of September 2024, teeth #18 and 48 were surgically removed under sedation and local anesthesia with Dentocaine 3.6 ml, 4%. The surgical protocol included triangular mucoperiosteal flap preparation and reflection at the site of tooth #48. The alveolar crest above the tooth crown was fenestrated using a surgical bone-cutting bur under continuous cooling with sterile saline. The tooth was extracted with a straight tooth elevator, the socket was debrided, rinsed with saline, and filled with a PRP gel (Figures 2, 3, 4). The flap was repositioned, adapted, and sutured with 5/0 monofilament polyamide material (Polyamide, Medipac, Greece) (Figure 5). For the extraction of tooth #18, an envelope flap was used. The rest of the surgical protocol was the same as that of tooth #48 (Figures 6, 7, 8).

PRP preparation

**Figure 9:**
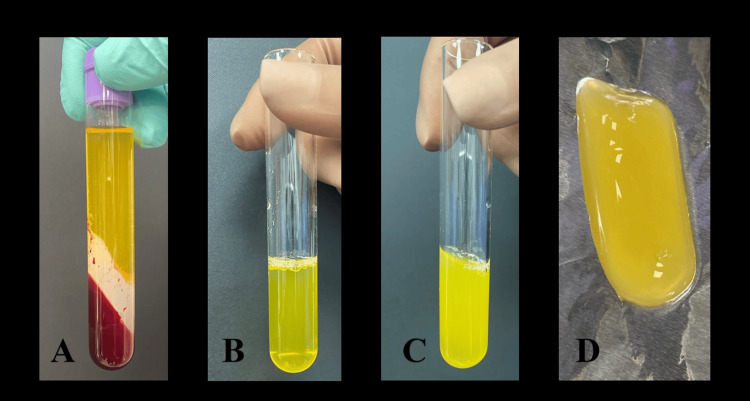
PRP preparation. A: after the first spinning; B: after the second spinning; C, D: after activation PRP: platelet-rich plasma

Venous blood (8 ml) was collected from the patient’s right median cubital vein and added to a vacutainer containing an anticoagulant and a separating gel. The blood was centrifuged twice, first at 3500 rpm/min for 10 minutes and then at 1900 rpm/min for five minutes. The so-called “buffy coat” (the layer rich in platelets and white blood cells) and part of the platelet-poor plasma in a total volume of 3 ml were transferred to a sterile tube and activated by 1 ml calcium gluconate (95.5 mg/ml) [7]. The PRP gel was formed for 20 minutes (Figure 9).

Postoperative care

The patient was given instructions for a soft diet and strict oral hygiene. Clindamycin 300 mg twice daily for six days, a probiotic, a non-steroidal anti-inflammatory drug (Ibuprofen 400 mg two to three times daily if necessary), and 0.12% chlorhexidine mouthwash, EluPerio (two to three times daily for 15 days), were prescribed.

The follow-up visits were on days one, two, and three to evaluate pain, facial edema, and trismus, and on day seven to remove stitches.

Second surgical intervention

Five weeks after the first surgery, teeth #28 and 38 were surgically removed under sedation and local anesthesia with Dentocaine 3.6 ml, 4%. The methodology fully repeated the above-described surgical protocol with only one difference: the post-extraction sockets were not filled with PRP but with hemostatic sponges instead.

Postoperative care

The patient was given the same instructions, and the same analgesics, mouthwash, and probiotics were prescribed. This time, Lymphomyosot solution, 20 drops three times a day for 10 days, and Azithromycin 500 mg, once daily for six days, were prescribed. The follow-up visits were on days one, two, and three to evaluate the postoperative sequelae and on day seven for suture removal.

Pain, facial edema, and trismus assessment

The post-extraction pain, facial edema, and trismus were assessed on the first, second, and third postoperative days. The pain was evaluated using the Visual Analogue Scale (VAS) and the analgesic intake.

**Figure 10:**
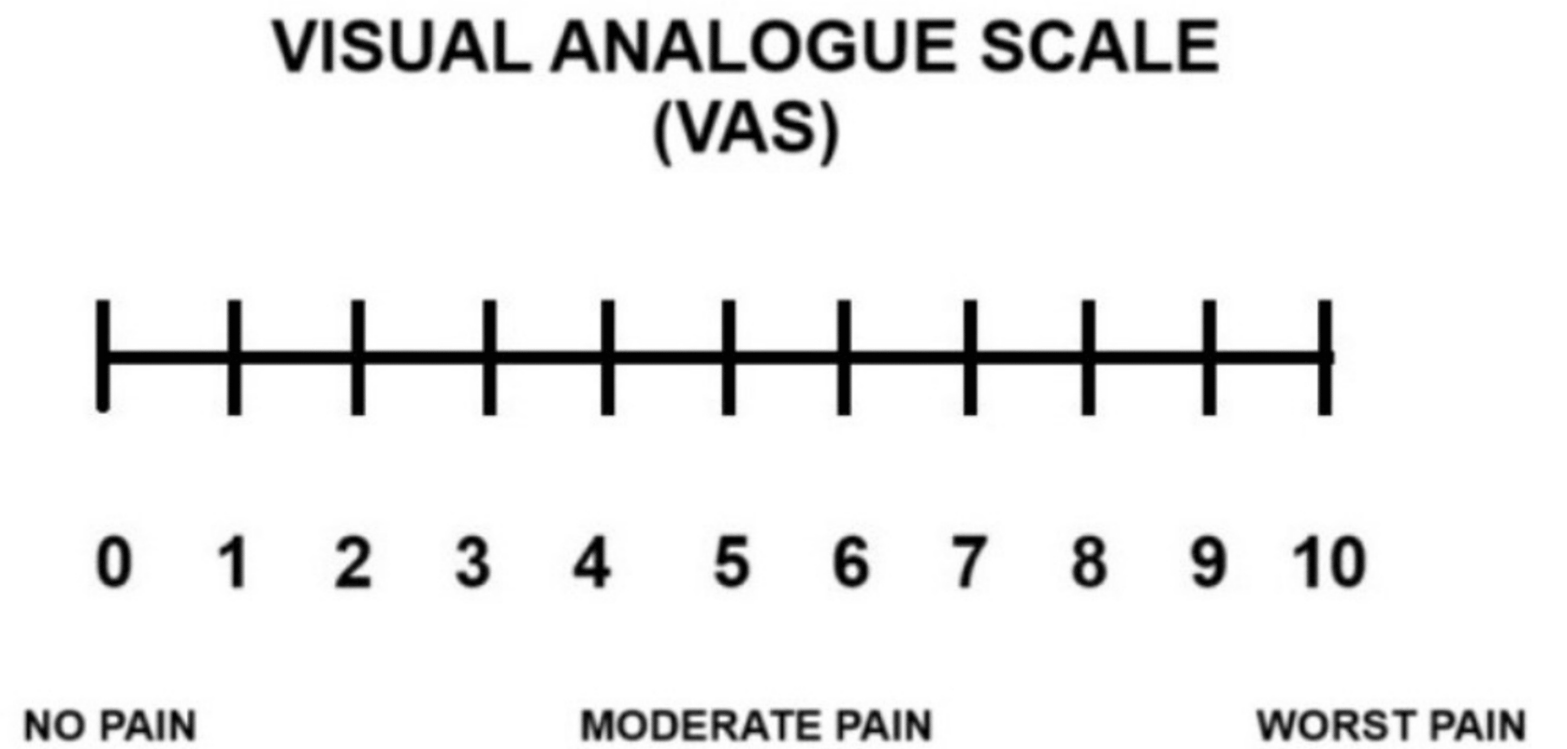
Visual analogue scale for pain evaluation.

The VAS assessment is usually performed by giving the patient a piece of paper with a horizontal line drawn, numbered from 0 (no pain) to 10 (severe pain), and the patient defining the pain intensity (Figure 10). Postoperative pain is usually controlled by non-steroidal anti-inflammatory drugs, opioids, and non-opioid analgesics. Analgesic intake is another indirect method to measure postoperative pain.

Normal mouth opening varies between 40 and 60 mm (two to three fingers’ width). Individual variations must also be taken into account. In addition, pain can trigger an instinctive or reflex limitation in the opening, and the clinicians should be able to establish the real etiology. Mouth opening restrictions (trismus) are evaluated by measuring the interincisal opening using a caliper. Grade 0 is registered when the distance is ˃2.5 cm; grade I is 2.0-2.5 cm; grade II is 1-2 cm; grade III is <1. Grades 0 and I are considered mild trismus, grade II moderate, and grade III severe trismus [8].

**Figure 11:**
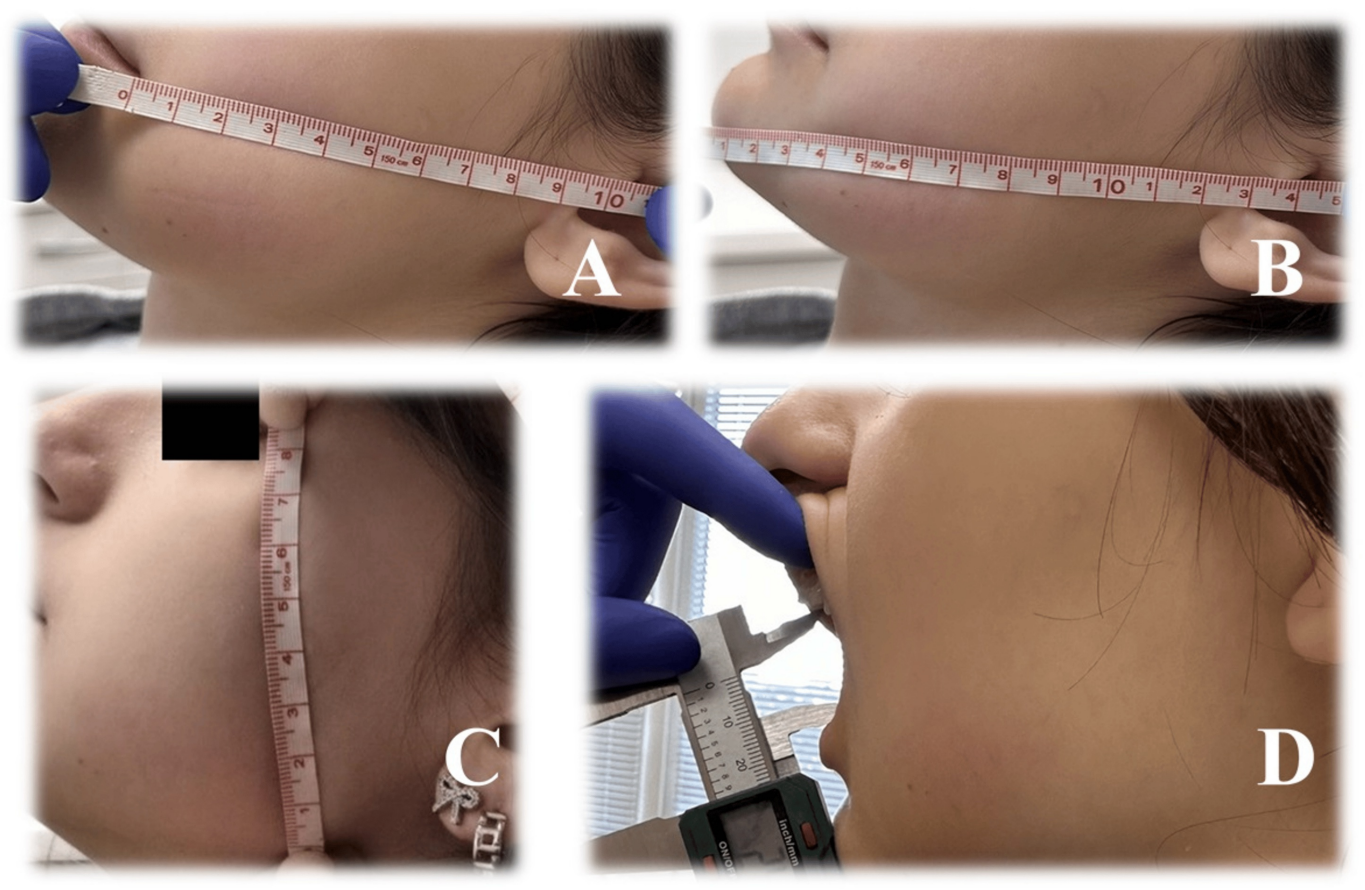
Swelling and trismus measurement. A: the distance from the tragus to the mouth corner (AC); B: the distance from the tragus to the most prominent point of the chin (AD); C: the distance from the lateral eye canthus to the lowermost point of the lower jaw angle (BE); D: mouth opening.

**Figure 12:**
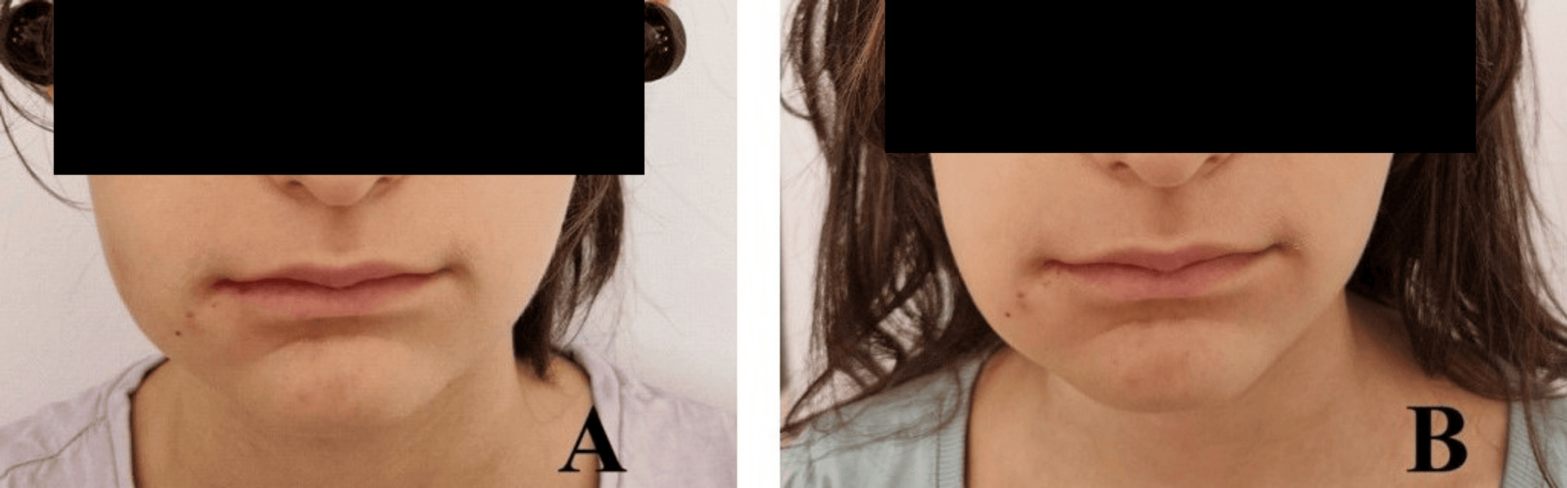
Facial edema on the second (A) and third (B) postoperative days after removal of the right wisdom teeth.

**Figure 13:**
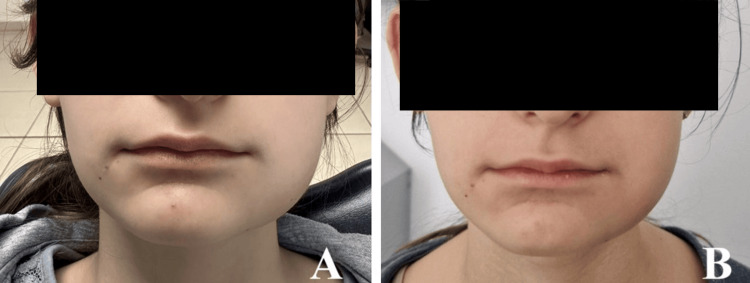
Facial edema on the second (A) and third (B) postoperative days after removal of the left wisdom teeth.

The post-extraction swelling was assessed by measuring the following distances: AC - from the tragus (A) to the mouth corner (C); AD - from the tragus to the most prominent point of the chin (D); BE - from the lateral eye canthus (B) to the lowermost point of the angle of the lower jaw. The facial size is calculated by the formula x = [(a + b)/2 + c]/2. The formula for the facial swelling is (x1 - x0)/ x0. 100%, with x1 being the postoperative facial size and x0 being the preoperative facial size. Facial edema is assessed using the following scale: Class 0 - facial edema area < 3%; class I - 3-6%; class II - 6-12%; class III > 12% (Figures 11, 12, 13) [8].

The measurements of the post-extraction pain, facial swelling, and trismus are presented in Table 1.

**Table 1:** Post-extraction pain, facial edema, and trismus measurements. PRP: platelet-rich plasma; AC: the distance from the tragus to the mouth corner; AD: the distance from the tragus to the most prominent point of the chin; BE: the distance from the lateral eye canthus to the lowermost point of the angle of the lower jaw; X_1_: postoperative facial size; X_0_: preoperative facial size; FE: facial edema; IIO: interincisal opening; IIO_R_: after the extraction of the right wisdom teeth; IIO_L_ :after the extraction of the left wisdom teeth; VAS: visual analogue scale; N/A: not applicable

Measurements		Day 0	Day 1	Day 2	Day 3
Right-side facial edema (PRP)		AC = 10.5 mm	AC = 10.6 mm	AC = 10.7 mm	AC = 10.6 mm
		AD = 13.2 mm	AD = 13.3 mm	AD = 13.5 mm	AD = 13.3 mm
		BE = 8.7 mm	BE = 8.7 mm	BE = 8.8 mm	BE = 8.7 mm
		X = 10.275	X_1_ = 10.325	X_1_ = 10.45	X_1_ = 10.325
		N/A	FE = 0.489% (Class 0)	FE = 1.7% (Class 0)	FE = 0.486% (Class 0)
Left-side facial edema (Lymphomyosot)		AC = 10.5 mm	AC = 10.6 mm	AC = 11.5 mm	AC = 10.7 mm
		AD = 13.2 mm	AD = 14.0 mm	AD = 14.9 mm	AD = 14.4 mm
		BE = 8.6 mm	BE = 9.1 mm	BE = 10.0 mm	BE = 9.2 mm
		X = 10.225	X_1_ = 10.7	X_1_ = 11.6	X_1_ = 10.875
		N/A	FE = 4.64% (Class I)	FE = 13.45% (Class III)	FE = 6.35% (Class II)
Mouth Opening	Before extraction	IIO = 37.2 mm	N/A	N/A	N/A
	After the extraction of the right wisdom teeth (PRP)	N/A	IIO_R_ = 25.2 mm (Grade 0)	IIO_R_ = 25.4 mm (Grade 0)	IIO_R_ = 30.5 mm (Grade 0)
	After the extraction of the left wisdom teeth (Lymphomyosot)	N/A	IIO_L_ = 24.1 mm (Grade I)	IIO_L_ = 24.3 mm (Grade I)	IIO_L_ = 30.1 mm (Grade 0)
Pain Score and Analgesic Intake	After the extraction of the right wisdom teeth (PRP)	N/A	VAS = 0 No Analgesic Intake	VAS = 0 No Analgesic Intake	VAS = 0 No Analgesic Intake
	After the extraction of the right wisdom teeth (Lymphomyosot)	N/A	VAS = 1 No Analgesic Intake	VAS = 0 No Analgesic Intake	VAS = 0 No Analgesic Intake

## Discussion

Postoperative pain, edema, and trismus are common non-infectious sequelae following third molar extraction. Infectious complications include dry socket, osteomyelitis, fistulae, and lymphadenopathy. Pain control is the main goal of postoperative care because it not only influences the patient's quality of life but also can impair tissue oxygenation and, thus, the healing process. Pain assessment is subjective and can be measured by the Visual Analogue Scale (VAS), the Numerical Rating Scale, the Wong-Baker Faces Pain Rating Scale, etc. [9].

Facial edema can be measured using stereophotogrammetry, tomography, ultrasound, sonographic examinations, photo-documentation (indirect measurements), and three-dimensional cast evaluation.

In this case report, the VAS assessment and analgesics intake were registered to identify the postoperative pain. The postoperative edema was assessed by measuring and calculating the facial size before and after the extraction and using a formula for facial swelling. The trismus was measured using a caliper. The results from this study demonstrate that PRP as a socket-filling material reduced postoperative facial edema in the first three days after the extraction (facial edema class 0 on days one, two, and three) and showed superior results than Lymphomyosot (facial edema class I, III, and II on days one, two, and three, respectively). PRP demonstrated a better effect on the postoperative mouth opening (grade 0 on days one, two, and three) than the homeopathic medication (grade 1 on days one and two and grade 0 on day three). As for pain relief, both methods demonstrated similar qualities. The patient reported no pain except for a slight discomfort on the first postoperative day when Lymphomyosot was used (VAS=1). No analgesic intake after both surgeries was reported.

Various studies advocate the application of autologous platelet concentrates, such as PRP and platelet-rich fibrin, for tissue regeneration and reduction of postoperative complications after third molar extraction. The rationale for their use is bone density improvement, accelerated soft tissue healing, and pain reduction [10,11,12,13,14]. For a more accurate assessment of bone fill and bone density, CBCT is preferred due to its superiority over two-dimensional radiographs [15,16]. The method has been regarded as the gold standard in evaluating the alveolar crest [17,18,19,20].

To the best of the author’s knowledge, the application of the multicomponent drug Lymphomyosot after oral surgery procedures has not been documented in the literature. It is a plant- and mineral-based medication, the indications of which, according to the manufacturer, include postoperative swelling reduction. As it is a homeopathic drug, it can be used for children undergoing third-molar extractions. Experimental models have advocated its effect on postoperative swelling and wound closure [21].

At the same time, PRP has recently stated its impressive potential as a regenerative material [22,23,24]. In terms of efficacy, it ranks alongside other well-studied biomaterials [25,26].

This study demonstrates the superior properties of PRP in reducing postoperative edema and trismus while PRP and Lymphomyosot gave similar results regarding pain control. Further case reports and randomized clinical trials would be beneficial in evaluating the effects of both methods. Split-mouth studies comparing Lymphomyosot to unassisted socket healing as a control should determine if the medication is indicated for third molar surgery.

## Conclusions

Pain, facial edema, and trismus are the most common postoperative sequelae after third molar extractions. Different techniques have been suggested to reduce their severity, including antibacterial drugs, corticosteroids, non-steroidal anti-inflammatory drugs, local antiseptics, cryotherapy, laser therapy, and autologous platelet concentrates. This case report compares the application of platelet-rich plasma and the combined medication Lymphomyosot to reduce the aforementioned post-surgical sequelae. It was evident that PRP had superior qualities to Lymphomyosot in terms of postoperative edema and trismus. Further research is necessary to evaluate their effect for these purposes.
